# Nerve Growth Factor: A Dual Activator of Noradrenergic and Cholinergic Systems of the Rat Ovary

**DOI:** 10.3389/fendo.2021.636600

**Published:** 2021-02-25

**Authors:** Agustin Benitez, Raul Riquelme, Miguel del Campo, Camila Araya, Hernan E. Lara

**Affiliations:** Centre for Neurobiochemical Studies in Neuroendocrine Diseases, Laboratory of Neurobiochemistry, Faculty of Chemistry and Pharmaceutical Sciences, Universidad de Chile, Independencia, Chile

**Keywords:** nerve growth factor, acetylcholine, norepinephrine, polycystic ovary syndrome, follicular development

## Abstract

The functioning of the ovary is influenced by the autonomic system (sympathetic and cholinergic intraovarian system) which contributes to the regulation of steroid secretion, follicular development, and ovulation. There is no information on the primary signal that activates both systems. The nerve growth factor (NGF) was the first neurotrophic factor found to regulate ovarian noradrenergic neurons and the cholinergic neurons in the central nervous system. The aim of this study was to determine whether NGF is one of the participating neurotrophic factors in the activation of the sympathetic and cholinergic system of the ovary *in vivo* and its role in follicular development during normal or pathological states. The administration of estradiol valerate (a polycystic ovary [PCO] phenotype model) increased norepinephrine (NE) (through an NGF-dependent mechanism) and acetylcholine (ACh) levels. Intraovarian exposure of rats for 28 days to NGF (by means of an osmotic minipump) increased the expression of tyrosine hydroxylase and acetylcholinesterase (AChE, the enzyme that degrades ACh) without affecting enzyme activity but reduced ovarian ACh levels. *In vitro* exposure of the ovary to NGF (100 ng/ml for 3 h) increased both choline acetyl transferase and vesicular ACh transporter expression in the ovary, with no effect in ACh level. *In vivo* NGF led to an anovulatory condition with the appearance of follicular cysts and decreased number of corpora lutea (corresponding to noradrenergic activation). To determine whether the predominance of a NE-induced polycystic condition after NGF is responsible for the PCO phenotype, rats were exposed to an intraovarian administration of carbachol (100 μM), a muscarinic cholinergic agonist not degraded by AChE. Decreased the number of follicular cysts and increased the number of corpora lutea, reinforcing that cholinergic activity of the ovary participates in controlling its functions. Although NGF increased the biosynthetic capacity for ACh, it was not available to act in the ovary. Hence, NGF also regulates the ovarian cholinergic system, implying that NGF is the main regulator of the dual autonomic control. These findings highlight the need for research in the treatment of PCO syndrome by modification of locally produced ACh as an *in vivo* regulator of follicular development.

## Introduction

Many studies have shown that ovary function is controlled by the sympathetic nervous system regulating steroid secretion, follicular development, and ovulation (1). Sympathetic nerves communicate with the ovary in two ways: the superior ovarian nerve with fibers localized around the follicles regulates steroid secretion and follicular development, and the ovarian plexus nerve mainly supplies innervation to blood vessels (2, 3). In addition, recent evidence supports the presence of an intraovarian cholinergic system located in granulosa cells (GCs) and involved in the development of ovarian follicles and ovulation (4–6). We have previously shown that the intraovarian cholinergic system mainly participates in the control of follicular development ovulation and atresia of antral follicles (6). Apparently, both noradrenergic and cholinergic systems regulate ovarian functions, probably working together or participating in a balanced way to regulate ovary function, similar to the autonomous regulation of many internal organs of the body (6). Their function is likely linked to maintaining the homeostatic condition of the organ, especially when the other part of the neuroendocrine axis (mainly gonadotropin-dependent control of the ovary) is being modified. In this sense, polycystic ovary syndrome (PCOS), the most frequent ovarian pathology causing infertility in women, is characterized by profound changes in follicular development, resulting in ovarian steroid secretion. In this condition, both neuroendocrine and nervous dysfunction have been observed in many other changes related to the metabolic and cardiovascular events associated with the syndrome (7). Due to the multitude of effects associated with PCOS, studies using animal models of the PCO phenotype are important. Recent studies have found that sympathetic stress, such as chronic exposure to cold (4°C for 3 h each day for 4 weeks), activates not only the sympathetic nerve fibers of the ovary but also the intraovarian cholinergic system (6). However, it is not known which primary signal activates both systems. The nerve growth factor (NGF) was the first neurotrophic factor found to regulate ovarian noradrenergic neurons (8). NGF is one of the most important factors in the regulation of cholinergic neurons in the central nervous system (9, 10), but there is limited information on its action in the ovary. If this is correct, we either can suggest that NGF acting on sympathetic nerves increases NE in the ovary by a direct effect of NGF or induced by stress (11, 12) and can participates in the development of the PCO phenotype in rat. It probably acts increasing the ACh concentration whose participation in ovary physiology is just recently been considered (4, 13); much less is know in pathological conditions such as the PCO phenotype in rat.

Recent evidence supports an additional role of NGF, not as a neurotrophic factor, but rather as a factor that regulates follicular development, affecting the survival or death of the follicular population (14). However, it is not known whether the primary actions are mediated by the NGF acting on nerve activity or NGF directed to the GCs to regulate follicular growth or death during development. Interestingly, NGF and TNF-alpha are part of a feedback loop similar to that associated with the inflammatory response, a common mechanism associated with ovary function (15).

Previous studies on the actions of NGF and norepinephrine on the ovary have shown that NGF’s action on follicular development is not only related to nerve activity but also involved in the control of ovarian follicular cells alongside the cholinergic system. Thus, the main aim of this study was to determine whether NGF is one of the neurotrophic factors involved in the activation of the cholinergic system of the ovary *in vivo*. In this work, we present data on a common neurotrophic mechanism acting on noradrenergic neurons and on ACh-producing cells to balance of the autonomic tone of the organ.

## Methods

### Animals and Experimental Design

We studied the effect of NGF on noradrenergic and cholinergic system in the rat ovary. Thus, we divided the experiments in two: 1. Studies *in vivo* in which we induced increase in noradrenergic transmission in the ovary by an NGF-mediated EV effect on the activity of the neurons in the ovary (16, 17). 2. the other experiment was chronic *in vivo* exposure by intrabursal administration of NGF to the ovary (11, 16). Once we determined the *in vivo* effect, we studied the effect of NGF *in vitro* to verify for a local effect of NGF on noradrenergic and cholinergic markers. After we defined the role of NGF in the activity of the noradrenergic or cholinergic biochemical markers, we analyzed the reproductive function and follicular development in the NGF treated rats. To differentiate from the cholinergic effect we also analyzed the role of a cholinergic muscarinic agonist Carbachol chronically administrated to the ovary on the follicular dynamic.

A total of 25 female Sprague–Dawley rats were used in this study: Six prepuberal (80–90 g) and 19 adult (250–300 g) animals (**Table 1**). All animals were housed in a maintenance room at a temperature of 20°C with light–dark cycles (12:12 h). The animals were provided food and water *ad libitum*. The estrous cycle of the adult rats was monitored *via* daily vaginal smears observed under a light microscope, as previously described (6, 19). The number of cycles was estimated as the regular passage from proestrus (P) to estrus (E), followed by diestrus (D). Control (sham) rats had regular 4-day estrous activity (Hubscher et al., 2005; Paccola et al., 2013). Ovaries from rats treated with estradiol valerate (EV) (intramuscular [i.m.] single dose, 10 mg/kg) were used. Bioethical regulation suggest to use tissue from other experiments previously published, we used one ovary of a previous study (18), that were stored at −80°C for ACh determination. At the end of the experiments, the rats were euthanized by decapitation, and the ovaries and plasma were collected. Decapitation was performed according to the AVMA Guidelines for the Euthanasia of Animals (2020 Edition) (20) by a specialized personnel. The study was also approved by the Bioethics Committee of the Faculty of Chemistry and Pharmaceutical Sciences at the University of Chile (Protocol number: CBE2017-14 to AB and CBE2017-05 to HL) and complied with the National guidelines (CONICYT Guide for the Care and Use of Laboratory Animals).

**Table 1:** Experimental groups used for the *in vivo* and *in vitro* studies.

*IN VIVO* STUDIES							
**1.-EV adm**.	**N**	**Age (days)**	**Days treatment**	**Biochemical studies ovary**	**Reprod function**	**Ovarian morphology**	**Origin of tissue**
10mg/kg i.m.	5	24 days old	30 days	ACh	———	————	Del Campo et al. (1)
Sham (sesame oil)	5	24 days old	30 days	ACh	———	————	Del Campo et al. (1)
**2.- NGF adm**. 100uM minipump	5	3-3.5 month old	30 days	TH WB ACh concentration AChase WB	Estrous cycle	Morphometry	Present work
Sham (saline)	5	3 - 3.5 month old	30 days	TH WB ACh concentration AChase WB	Estrous cycle	Morphometry	Present work
**3.- Carbachol adm**. 100uM minipump	4	3 - 3.5 month old	30 days	—————–	Estrous cycle	Morphometry	Present work
Sham (saline)	5	3 - 3.5 month old	30 days	—————–	Estrous cycle	Morphometry	Present work
***IN VITRO* STUDIES**							
Control rats	6	Prepuberal (two half)	Control medium	Half ovary Half ovary	ACh, mRNA		Present work
Ovaries cut in half and used		Prepuberal (two half)	NGF(100 ng/ml)	Half ovary Half ovary	ACh mRNA		Present work

(1) Ovaries were stored frozen at -80C from an experimental serie done for the paper of (18).

### *In Vivo* NGF and Varbachol Administration Studies

Nineteen adult female rats were randomly assigned to either the sham group (control group) (n = 5) or NGF group (n = 5) for NGF studies, and sham group (control group) (n = 5) or carbachol group (n = 4) for carbachol studies (**Table 1**). The animals were anesthetized with an intramuscular dose of ketamine 60 mg/kg and xylazine in 10 mg/kg solution under aseptic conditions. To eliminate the possible contribution of the contralateral ovary to steroidogenesis, all sham, NGF- and carbachol-treated animals were subjected to unilateral ovariectomized (ULO) at the moment of the minipump implant (19), performed as previously reported (16).

To eliminate the possibility of a confounding effect of ovary hypertrophy derived from the ULO, all sham and experimental rats, were subjected to hemiovariectomy. Briefly, a transverse midlumbar incision, 1.5 cm long, was made in the flank area on one side of the animal to obtain access to the ovarian bursa. The ALZET osmotic minipump ([0.25 μl/h] Model 2004; Alza Corp. Palo Alto, CA, USA) was connected to the underlying bursa of the left ovary with SILASTIC 0.64 mm ID × 1.19 mm OD CAT 508-003 (Dow Corning Corp, Midland, MI, USA) tubing for 28 days. The treatment was performed as follows:

a) Animals in the NGF group were implanted with osmotic minipumps for intraovarian NGF delivery at a concentration of 100 ng/ml in saline (catalog number N-100; Alomone Labs, Jerusalem, Israel).
b) Animals in the carbachol group were implanted with osmotic minipumps for intraovarian delivery at a concentration of 100 µM in saline (catalog number 212385-M; Calbiochem, Sigma Chemicals, St Louis, MO, USA). Carbachol, is a well-known non-specific muscarinic cholinergic agonist that is not degraded by AChE
c) For the sham group, animals were subjected to surgery and implanted with the cannula filled with saline, the solvent for both drugs, but not the osmotic minipump.

After 28 days, the rats were euthanized and the ovary and trunk blood were collected for analysis. The position of the minipump and the cannula was inspected to verify that they were in place after the two or one month procedure. A picture of both is shown in supplementary data. The ovaries were cut in half, and one half was fixed with Bouin’s fluid for morphometric analysis. The other half was cut again in half, and each half was stored at −80°C for ACh determination or western blot analysis for NGF studies.

### *In Vitro* NGF Studies

To study the effects of NGF on ACh production in the ovaries, we used six prepuberal rats (**Table 1**). We used prepuberal rat ovaries because, at this age, there is no ovulation and no corpora lutea; thus, we can study the effect of NGF mainly in GCs (21, 22). The rats were euthanized, and both ovaries were removed through an anterior incision in the midline of the abdomen. The ovaries were halved (2 ovaries = 4 halves per animal), and each half was incubated for 3 h at 37°C in 1.0 ml of Krebs-bicarbonate-albumin buffer (NaCl, 118.6 mM; KCl, 4.7 mM; KH_2_PO_4_, 1.2 mM; ascorbic acid, 100 μg/ml; NaHCO_3_, 0.15 M; CaCl_2_, 25 mM; albumin, 0.1 mg/ml; glucose, 11.2 μg/ml), under 95% oxygen and 5% CO_2_. For each condition, the animals were randomly divided into groups of six. One half was incubated only in Krebs-bicarbonate buffer (the control group), and the other half was incubated with NGF at 100 ng/ml (catalog number N-100, Alomone Labs, Jerusalem, Israel). The concentration of NGF used has been previously demonstrated to be sufficient to increase choline acetyl transferase (ChAT) protein levels in human GCs and ACh levels in bovine luteal cells (9, 10). After incubation, the ovaries were stored at −80°C for mRNA extraction or ACh determination at a later date.

### Quantification of Intraovarian Levels of ACh and AChE Activity

The ovary was homogenized in 10 volumes of PBS in ice. ACh and AChE activity was determined in the homogenate using the Amplex^®^ ACh/AChE assay kit (Invitrogen, Carlsbad, CA, USA) according to the instructions recommended by the provider as previously described (4, 6). The results represent the total amount of ACh in μmol per ovary and AChE activity in U per ovary (where one U is defined as the amount of enzyme that hydrolyzes 1.0 µmole of ACh to choline and acetate per minute at pH 8.0 at 37°C, as indicated by the manufacturer). The minimal detectable value for AChE was 0.002 UI/ml and for ACh was 0.3 μM (range, 0.3 μM to 100 μM).

### Western Blot Analysis

For western blotting, the ovary was homogenized in 10 volumes of RIPA buffer (1% NP40, 0.5% sodium deoxycholate, and 0.1% SDS in PBS; just before use, 10 μl of the following mixture [10 mg/ml stock solution of PMSF; aprotinin and sodium orthovanadate] was added) in the presence of Complete Mini EDTA-Free Protease Inhibitor Cocktail (Roche, Basel, Switzerland). Proteins were quantified by the Bradford method, and 50 μg was run on a 10% polyacrylamide gel. Proteins were transferred to nitrocellulose, blocked with 5% milk for 1 h, and incubated with an antibody that recognizes all the isoforms of AChE (A-11; Santa Cruz Biotechnology, Dallas, TX, USA) at a dilution of 1:3,000 overnight or with tyrosine hydroxylase (MAB5280; Merck Millipore, Burlington, MA, USA) at a dilution of 1:1,000 overnight. As an internal control, we used GAPDH (G9545; Sigma Chemicals, St Louis, MO, USA) at a dilution of 1:40,000 for 1 h. The secondary antibodies used were goat anti-mouse IgG Fc (HRP) (catalog number 31430; Waltham, Massachusetts, USA) at 1:10,000 dilution for 1 h, and goat anti-rabbit IgG Fc (HRP) (ab97200; Abcam, Cambridge, United Kingdom) at 1:10,000 dilution for 1 h. The antibody complexes were detected by chemiluminescence using an EZ-ECL Enhanced Chemiluminescence Detection Kit (Biological Industries, KBH, Israel). Chemiluminescence was captured using a G-Box Syngene system (Syngene Headquarters, MD, USA).

### Morphometric Analysis

The halved ovaries were fixed in Bouin’s fluid, embedded in paraffin, cut into 6-μm sections, and stained with hematoxylin and eosin. Morphometric analyses of whole ovaries were performed according to the method of (23) with modifications described previously (16), using n = 5 ovaries for the sham group, n = 5 ovaries for the NGF group, and n = 4 ovaries for the carbachol group. We used the following classification: primordial follicles had one oocyte surrounded by flattened GCs. Primary follicles had one layer of cubical GCs, and secondary follicles had no antral cavity but two or more layers of GCs. Antral follicles were those with more than three healthy GC layers, the antrum, and a clearly visible nucleus of the oocyte. Atretic follicles had more than 5% of the cells with pyknotic nuclei in the largest cross-section and exhibited shrinkage and occasional breakdown of the germinal vesicle. Precystic follicles were large follicles with or without oocyte, containing four or five plicated layers of small, densely packed GCs surrounding a very large antrum with an apparently normal thecal compartment. Cystic follicles were devoid of oocytes and displayed a large antral cavity, a well-defined thecal cell layer, and a thin (mostly monolayer) GC compartment containing apparently healthy cells. All abnormal follicular structures were grouped as cystic structures.

### Plasma Levels of Steroid Hormones

Plasma levels of steroid hormones progesterone (P4), androstenedione (Δ4), testosterone, and estradiol (E2) were measured. Serum levels of P4, Δ4, testosterone, and E2 were determined by enzyme immunoassay (EIA), following the manufacturer’s instructions (Alpco Diagnostic, Windham, NH, USA). Intra and interassay variations were less than 5% for P4, less than 10% for Δ4, less than 6% for testosterone, and less than 5% for E2, and the minimal detectable values were 0.1 ng/ml, 0.04 ng/ml, 0.02 ng/ml, and 10 pg/ml, respectively.

### Real-Time Polymerase Chain Reaction (qPCR)

Total RNA was extracted as described previously (24) from the halved ovary incubated *ex vivo*. The primers used are listed in **Table 2**. A BLAST search was performed to determine the specificity of the sequences. The PCR reaction mix contained 10 μl of Brilliant II SYBR Green QPCR Master Mix (Agilent Technologies, Inc., California, USA), 0.01 μM of each GAPDH primer, 0.1 μM of each ChAT primer or 0.1 μM of each vesicular ACh transporter (VAChT) primer, 2 µg of cDNA, and sterile water for a final volume of 20 μl. PCR reactions were performed using the IQ5 real-time thermocycler (Bio-Rad) under the following conditions: 95°C for 20 s, 60°C for 20 s, 72°C for 20 s, and a final extension at 72°C for 10 min. All samples for RT-qPCR analysis were run in triplicate (with no reverse transcriptase control as a negative control), and the mean values were used to determine the mRNA levels. Relative quantifications of ChAT and VAChT mRNA were performed using GAPDH mRNA as a housekeeping gene.

**Table 2:** Primers used for polymerase chain reaction (PCR) amplification.

Gen	Sequences		Access number	Sequence reference
ChAT	*forward*	5′- CTGGATTTCATTGTTTATAAGTTTGACAAC-3′	XM_00106152	(25)
	*reverse*	5′- CTGGAGGGCCACCTGGAT-3		
VAChT	*forward*	5′- GCCACATCGTTCACTCTCTTG-3′	X80395	(26)
	*reverse*	5′- CGGTTCATCAAGCAACACATC-3′		
GAPDH	*forward*	5′- GATGCCCCCATGTTTGTGAT -3′	NM_017008.4	(27)
	*reverse*	5′- GGTCATGAGCCCTTCCACAAT-3′		

### Statistical Analysis

The data are expressed as the mean ± SEM. Statistical analyses were performed using Prism GraphPad 6 (GraphPad Software, San Diego, California, USA). To examine statistical differences between the two groups, we used Student’s *t* and the Mann-Whitney tests, as described below. To analyze differences between proportions, we used the chi-square test. To determinate normal distribution of our data, we used the Shapiro-Wilk normality test.

The number of animals for all experiments was calculated as the minimum number of animals according to the variability of the experimental procedures and the intrinsic variation between them. The minimum number of animals was calculated according to the following equation (28):

$$n=\frac{2{\left(Z\alpha +Z\beta \right)}^{2}\times S^{2}}{d}$$

where n is the number of animals for each condition, S = standard deviation, d = difference needed to obtain statistical significance, Zα = the probability of type I error (significance), and Zβ = the probability of type II error (power). In the experiments to determine ACh, and AChE activities and levels of plasma hormones, we proposed α = 0.05, the probability of finding a statistically significant difference was 0.05; β = 0.3, the probability of having a difference between the populations; the intrapopulation variation, was 0.2; and d, the smallest difference in the population, was 0.11. Thus, we obtained n = 4.5. Therefore, to obtain a statistically significant difference of p<0.05, we needed to use four or five animals per study group.

## Results

### Estradiol Valerate (EV) Increased Ovarian ACh Levels

A previous report showed that after 30 days of estradiol valerate (EV) exposure, there was an increase in NGF level in the ovary; 60 days after EV, there was also an increase in norepinephrine level (17). We found that ovarian ACh levels had also increased ([mean ± SEM] 5.0 ± 0.6 µmol/mg ovary for sham vs. 12.3 ± 3.1 µmol/ovary for EV-treated rats, p < 0.05, unpaired Mann-Whitney test, n = 5).

### Effect of *In Vivo* Intraovarian Exposure to NGF on Autonomic Neurotransmitter

**Figure 1:**
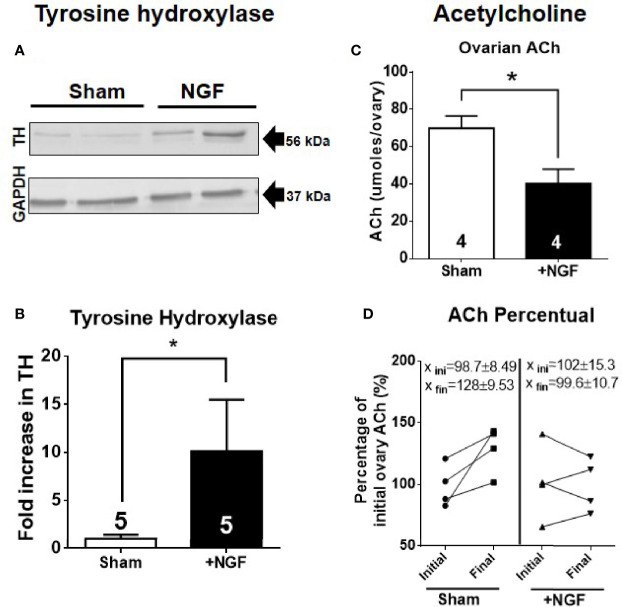
*In vivo* intraovarian nerve growth factor (NGF) administration increases tyrosine hydoxylase (TH) levels and decreases acetylcholine. **(A)** Western blot analysis of tyrosine hydroxylase (~56 kDa) and GAPDH in the ovary. Representative membranes of each protein are shown. **(B)** Bar charts show quantification of protein levels of TH compared to GAPDH control in each condition. Pixels were counted using ImageJ software (U.S. National Institutes of Health, Bethesda, Maryland, USA). **(C)** Decreased levels of ovarian ACh level. Unpaired Student’s t test. No change was found in ovary weight after 28 days of treatment between the sham and NGF groups (sham = 74.61 ± 8.189 mg; NGF = 52.78 ± 10.44 mg). **(D)** A decrease in percent ACh levels after NGF treatment relative to initial level – Initial ovary corresponds to contralateral ovary that was removed on day 0: Sham = 128 ± 9.53% vs. NGF = 99.6 ± 10.7%. *P < 0.05, Chi-square test. All values correspond to the mean ± SEM. *P < 0.05.

The concentration of NGF used would be sufficient to promote biological actions on the sympathetic neurons fibers that innervate the ovary (29–31). To ensure sympathetic activation, we determined tyrosine hydroxylase (TH) levels (MW: 56 kDa) by western blot analysis (**Figure 1A**). **Figure 1B** shows the quantification of protein levels relative to the mean of the sham group level. *In vivo* NGF treatment produced a 10-fold increase in TH.

We found a decrease in ovarian ACh levels per ovary after 28 days of stimulation with 100 ng/ml NGF (**Figure 1C**). No differences were found in ovarian weight (data not shown). When we compared the ACh levels of the ovaries between before and after NGF treatment (where the ovaries before treatment corresponded to the contralateral ovary removed before starting the osmotic minipump implantation or sham surgery) for each condition, we found a significant decrease in ACh in the NGF-treated ovaries (**Figure 1D**).

### Effect of *In Vivo* Intraovarian Exposure to NGF on Ovarian AChE Isoform and Enzyme Activity

**Figure 2:**
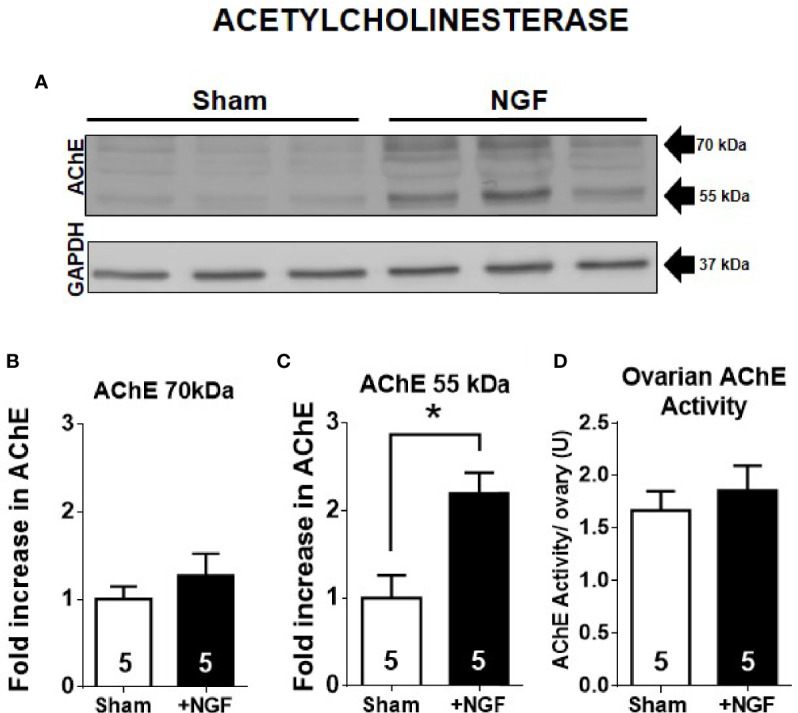
55 kDa-isoform AChE is increased by *in vivo* nerve growth factor (NGF) treatment. **(A)** Western blot analysis of AChE and GAPDH in the ovary. Representative membranes of each protein are shown, and two main bands at ~70 kDa and ~55 kDa were identified. GAPDH was used a loading control. **(B)** Bar chart shows quantification of protein levels of AChE 70 kDa-isoform. No significant change was found. Pixels were counted using ImageJ software. **(C)** Bar charts shows the quantification of protein levels of AChE 55 kDa-isoform. All values correspond to the mean ± SEM. **(D)** Ovarian AChE activity was not affected by *in vivo* administration of NGF. All values correspond to the mean ± SEM of n = 5 experiments, *P < 0.05, unpaired Mann-Withney test.

ACh levels are mainly regulated by cholinesterases. In the ovary, the main cholinesterase is AChE (32). Ovarian rat cells express two isoforms: AChE subtypes –R and –S (4). Therefore, to determine if the decrease in ovarian ACh levels is mediated by an increase in AChE, we analyzed its levels by western blot. In this sense, while AChE-R has a size of approximately 55 kDa (33–35), AChE-S has several posttranslational modifications, and its size could be 70, 60, and even 55 kDa (33–36). **Figure 2** shows the western blot analysis results for AChE, and we found two main bands at 70 kDa and 55 kD (**Figure 2A**). The total group data are presented in **Figures 2B, C**. *In vivo* NGF treatment produced a two-fold increase only in the AChE 55 kDa-isoform (**Figure 2C**). We used a monoclonal antibody that is targeted to the *N-*terminal (A-11, Santa Cruz Biotechnology), common to both isoforms, so we could not discriminate between AChE subtypes.

To determine the amounts of enzymatic proteins, especially the AChE 55kDa-isoform, we determined the ACh hydrolysis capacity of the ovary samples because this isoform is mainly present in the ovary and not butyryl cholinesterase (32). We did not find changes in enzyme activity between the NGF-treated and sham groups (**Figure 2D**). Therefore, the increase in AChE 55 kDa-isoform levels was not related to an increase in its catalytic activity.

### *In Vitro* NGF Incubation Increased ChAT and VAChT mRNA Levels But Not ACh Levels

**Figure 3:**
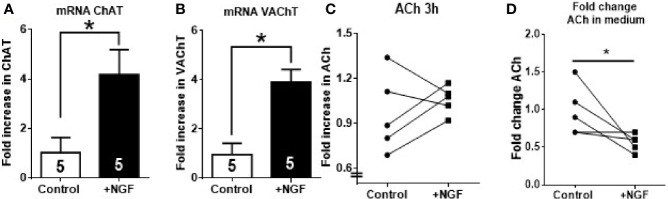
*In vitro* nerve growth factor (NGF) incubation increases ChAT and VAChT mRNA but decrease ACh in media. Half ovaries were incubated in Krebs buffer for 3 h: Control, incubated only in media; NGF, incubated with NGF at 100 ng/ml. **(A, B)** Fold increase in the mRNA expression of ChAT and VAChT after incubation with NGF. GAPDH mRNA was used as housekeeping gene. **(C)** A slight but not significant increase was found in ACh levels in 60% of animals. **(D)** ACh fold change in incubation media. All values are relative to control mean and, for each group, correspond to the mean ± SEM of n = 5 experiments. *P < 0.05 unpaired Mann-Withney test.

*In vivo* NGF treatment affects not only the ovarian cholinergic system but also extrinsic sympathetic fibers that innervate the organ as well as other factors that are present in a rat. To rule out all these extrinsic factors, **Figure 3** shows the effect of *in vitro* ovary culture treated with 100 ng/ml of NGF on ChAT and VAChT mRNA expression and ACh levels in rat ovary. Reverse transcription qPCR (RT-qPCR) studies showed that *in vitro* NGF incubation for 3 h produced a four-fold increase in ChAT and VAChT mRNA levels compared to the control condition (**Figures 3A, B**). AChE mRNA levels showed no changes (data not shown). A slight but not significant increase in ovarian ACh levels was observed in two-thirds of the samples after 3 h of stimulation (**Figure 3C**). Interestingly, ACh was reduced but only in incubation media.

### Effect of *In Vivo* NGF Exposure Administration on Estrus Cycle

**Figure 4:**
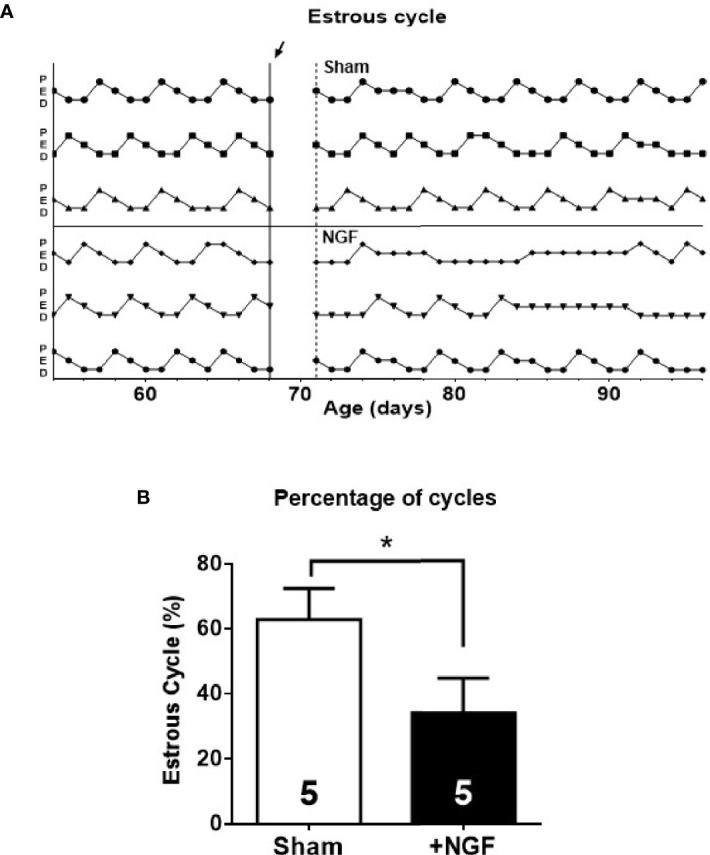
Estrous cyclicity was affected by *in vivo* nerve growth factor (NGF) administration. Sham, animals exposed to sham surgery; and NGF, animals exposed to 100 ng/ml of NGF locally delivered to the ovary by the means of an osmotic minipump. Animal weights after 28 days of treatment were: Sham = 311.3 ± 8.147 g vs. NGF = 298.3 ± 12.91 g (n.s). **(A)** Three representatives estrous cycle profiles before (14 days) and during treatment (28 days) for each condition are shown (*arrow* indicates osmotic minipump implantation or sham surgery). Vertical axis depicts different stages of the estrous cycle. **(B)** Each bar represents the number of estrus cycles estimated as the regular passage from proestrus (P) to estrus (E) followed by diestrus (D) over the observation days. All values correspond to the mean ± SEM of n = 5 animals. *P < 0.05, unpaired Student’s t test.

The exposure of the ovary to NGF during the 28-d disrupted the estrous cycle (**Figure 4A**), as previously described (11). Compared to the sham group, the treated group showed a significant decrease in the percentage of time of proestrus (NGF = 15.4 ± 2.2% vs. sham = 21.3 ± 1.3%; *P < 0.05) and an increase during estrus (NGF = 33.7 ± 3.1% vs. sham = 26.0 ± 1.7%; *P < 0.05). There was a significant decrease in the number of ovulatory estrous cycles (NGF = 25.0 ± 6.9% vs. sham = 64.1 ± 7.9%; *P ≤ 0.05) (**Figure 4B**). All of these changes were previously described (11)

### Ovarian Follicular Dynamic Was Altered by *In Vivo* NGF Administration

**Figure 5:**
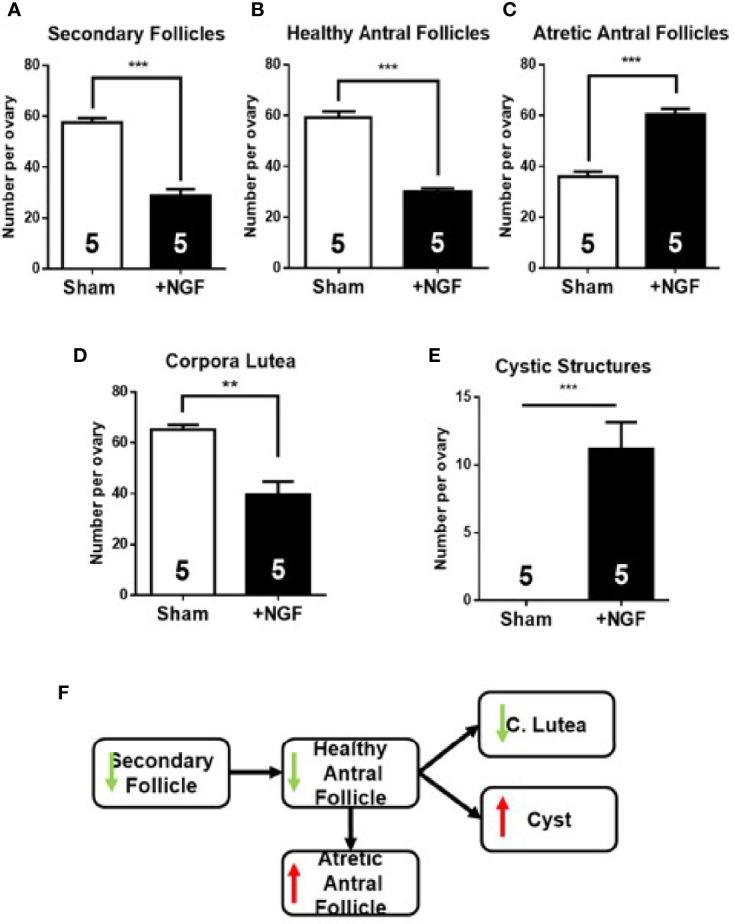
Morphometric analysis of ovaries after *in vivo* intrabursal exposure during 28-day with 100 ng/ml nerve growth factor (NGF). Altered follicular dynamic was found in NGF-treated animals: decrease in number of secondary **(A)** and healthy antral **(B)** follicles, an increase in number of atretic antral follicles **(C)**, minor increase in corpora lutea, **(D)** and appearance of cystic structures **(E)** is visible. **(F)** Summary of changes found in follicular development. All values correspond to the mean ± SEM (n = 5 for each group). **(A–C, E)** ***P < 0.001, **(D)** **P < 0.01, unpaired Student’s t test.

Morphometric analysis results of the ovary exposed to excess NGF for 28 days are shown in **Figure 5**. As previously described (11), important alterations in normal follicular development were found, with a reduction in the number of secondary follicles (**Figure 5A**) and healthy antral follicles (**Figure 5B**). Moreover, there was an increase in atretic antral follicles (**Figure 5C**), and a decrease in the number of corpora lutea (**Figure 5D**). NGF exposure for 28 days resulted in the appearance of cystic structures (**Figure 5E**). A decrease in the number of healthy antral follicles and an increase in atretic antral follicles, along with the appearance of cystic structures, have also been observed in mice and rats treated with an excess NGF (11).

### Plasma Concentration of Progesterone Reduced After Chronic NGF Administration

**Table 3** shows the plasma levels of ovarian steroids, progesterone, androstenedione, testosterone, and estradiol at the end of the experimental protocol. NGF treatment for 28 days led to a reduction in the progesterone levels. No changes were found in androstenedione, testosterone, and estradiol levels.

**Table 3:** Plasma concentration of progesterone, androstenedione, testosterone, and estradiol after NGF treatment.

	Sham	NGF-treated
Progesterone (ng/ml)	10.8 ± 1.9	4.5 ± 1.8 (*)
Androstenedione (ng/ml)	0.33 ± 0.2	0,29 ± 0.1
Testosterone (ng/ml)	0.32 ± 0.1	0.24 ± 0.1
Estradiol (pg/ml)	22.3 ± 2.6	26.3 ± 5.0

Results correspond to five animals in each condition and are expressed as mean value ± SEM. *P < 0.05, unpaired Student’s t test.

### *In Vivo* Carbachol Administration Promoted Follicular Development

**Figure 6:**
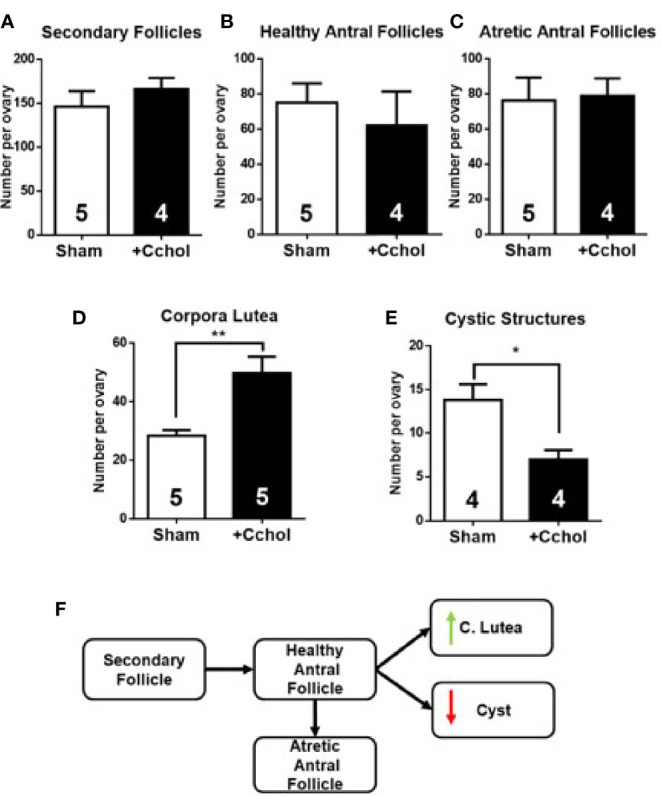
Morphometric analysis of ovaries after *in vivo* intrabursal exposure during 28-day with 100 uM Carbachol (Cchol). No significant changes in weight gain were observed between experimental groups: Sham, animals exposed to sham surgery; and carbachol (Cchol), animals exposed to 100 uM of Cchol locally delivered to the ovary by means of an osmotic minipump. Follicular development was enhanced: No changes in number of secondary **(A)**, healthy **(B)**, and atretic antral **(C)** follicles, but an increase in number of corpora lutea **(D)**, and a decrease in number of cystic structures **(E)** are visible. **(F)** Summary of changes found in follicular development. All values correspond to the mean ± SEM of n = 4 or 5 animals as shown in the figure. **(D)** **P < 0.01, **(E)** *P < 0.05, unpaired Student’s t test.

We found that *in vivo* NGF treatment increases intraovarian TH levels, reduces intraovarian Ach levels, and causes aberrant follicular development. To determine if the decrease in ACh mediates this enhanced response of NGF to the sympathetic nerves, we used chronic administration of carbachol, a muscarinic agonist that is not degraded by AChE. No changes in the estrous cycling activity was found (not shown). Morphometric analysis of the ovaries after carbachol exposure showed no changes in secondary and antral follicles, but increased numbers in the corpus luteum and a decrease in cystic structures (**Figure 6**), suggesting cholinergic promotion of the healthy pathway.

## Discussion

Recent evidence strongly suggests that when the balance between the sympathetic and cholinergic pathways is altered, this may lead to pathologic conditions, such as that induced by estradiol valerate treatment, a model that resembles PCOS (16, 37, 38). Sympathetic fibers innervate the ovary in the establishment of PCOS conditions. ACh is involved in several processes related to ovarian function, but how its ovarian production is regulated is unclear. Recently, we found that chronic sympathetic stimulation by stress stimulated the intraovarian cholinergic system (6). Here, we found that estradiol valerate produces an increase in ACh levels, similar to that observed after chronic cold stress. Our most important observation was the stimulatory effect of NGF *in vitro* on the increase in the metabolizing enzymes of ACh, and the effect was repeated after long-term *in vivo* treatment. The fact that it was not translated into changes in ACh led to an unbalanced sympathetic/cholinergic system, resulting in aberrant follicular development with a concomitant decrease in progesterone plasma levels. Since NGF regulator of the dual autonomic control, which is essential for maintaining the homeostasis of ovary function.

In sympathetic neurons, estradiol valerate treatment leads to an increase in ovarian noradrenaline levels and an ovarian phenotype similar to that in PCO (17, 39). After 30 days, estradiol valerate has been found to produce an increment in NGF and NGFR levels in rat ovary which is also associated with PCO phenotype in rats (16). The implant in the ovary of cells overexpressing NGF are involved in the development of PCO in mouse (14, 40). It is not surprising to found estrogen dependent changes in neurotrophin in sympathetic nerves and in cholinergic neurons because it has been amply demonstrated that estrogens converge with neurotrophin signaling pathways (41, 42). The increase in the biosynthetic enzymes for ACh indicate that NGF regulates ovarian ACh production. Previous reports also suggest that NGF stimulates ACh production (6, 9, 10). This increase could protect follicular development from the actions of an over-activated sympathetic pathway and chronic increase in ovarian noradrenaline, caused by the hyperinnervation of the organ (39, 40). To explore the effects of NGF on the activities of the enzymes involved in the biosynthesis and degradation of intraovarian ACh in the rat ovary, we utilized *in vivo* and *in vitro* approaches.

### *In Vivo* NGF Treatment Reduced ACh Levels and Enhanced Production of 55kDa-Isoform AChE

*In vivo* treatment with intrabursal NGF disrupted the estrous cycle, as demonstrated in previous studies that either grafted the ovary with cells overexpressing NGF (11) or used transformed cells producing NGF (11, 14). Locally delivered NGF was effective in activating sympathetic neurons, as evidenced by the increased expression of TH, the rate-limiting enzyme in the biosynthesis of NA. The ovaries of animals treated with NGF were hyperinnervated by catecholaminergic fibers, giving an enhanced sympathetic tone to the gland (16). Although NGF increased the activities of the enzymes involved in the biosynthesis of ACh, NGF unexpectedly decreased ACh levels, even with no changes in AChase activity.

When we compared the ovaries at the end of the treatment with NGF with the contralateral ovary collected at before the minipump installation, we found that the NGF-treated ovaries had significantly lower ACh levels per ovary than the non-treated ovaries. We do not have information about the release mechanism inside the GCs and whether it is regulated by other factors (43). However, in primary cultures of cholinergic neurons, NGF has been found to promote ACh release (vesicular as well as spontaneous) faster than enhancing ChAT activity, in a concentration- and time-dependent manner up to 10 days (43, 44). (6), proposed a model in which ACh was stored in vesicles inside the GC and would not be exposed to degradation by AChE. However, ACh, once released, does not act on muscarinic receptors to exert trophic action. Further research is needed to evaluate ACh release mechanisms in GC.

ACh levels are also regulated by cholinesterases. It has been reported that ovarian cholinesterase activity is mainly mediated by the ovarian AChE (32). AChE isoforms –S and –R have been recently identified in rat ovaries (4). It is known that they have different characteristics and distributions: isoform S is able to form multimers that join to anchoring proteins and bind them to the synaptic membrane, while AChE-R cannot form it and once secreted, it is soluble. In studies involving mice and humans, AChE-S has shown different molecular weights (55, 60, and 70 kDa) depending on posttranslational modifications (33–36). The molecular size of AChE-R was found to be 55 kDa in studies involving mice and humans (33–35). When we analyzed AChE by western blotting after NGF stimulation and found a 2-fold increase only in the 55 kDa-isoform. The monoclonal antibody (A11, Santa Cruz Biotechnology) that we used did not discriminate between both isoforms. Besides, it is known that AChE levels increase to compensate for the excess in ACh in the brain, such as the hippocampus and caudate nucleus (45, 46). However, in our study, there was no increase in ACh or AChE activity. Therefore, we thought that this increase in the mass of the protein might be related to AChE-R. In this sense, the action of stress or AChE inhibitor leads to alternative splicing that produces a large amount of AChE-R mRNA (46). However, AChE-R mRNA is less stable than AChE-S mRNA (47), and its expression is limited to the duration of its stimulus. Further research is needed to explore this hypothesis.

### NGF Increased ChAT and VAChT mRNA But Not ACh Production

Three hours of incubation of the ovary in the presence of NGF increased the level of ChAT mRNA. This change is in agreement with results in previous reports where 100 ng/ml of NGF stimulated ChAT production in bovine luteal cells and human GCs (9, 10). Although it has not been established that ACh is stored in vesicles, VAChT has been found to be expressed in GCs (21). Both proteins are expressed together in many neuronal models because they share the same transcriptional direction, since VAChT genes are located in the first intron of ChAT genes (48). We also found an increase in VAChT mRNA after NGF treatment; therefore, the effects related to the expression of proteins probably stimulated ACh production and storage. However, despite these promising results, when we analyzed ACh levels in our *in vitro* model, we found a slight but not significant increase in ACh levels in 66% of samples after 3 and 24 h of NGF treatment. Several factors were considered in our experimental protocol. First, we used prepubertal rat ovaries to rule out the interference of structures that do not produce ACh in rats, such as the corpus luteum (21, 49). Second, our model has been demonstrated to be useful for evaluating the ovarian effects of different neuropeptides, including NGF (19, 50, 51). Isolated ovaries eliminate the exogenous contributions from cholinergic and sympathetic neuronal fibers that innervate the organ. In the incubation medium, we found a decrease in ACh levels, but this may be related release or increase in AChE activity. In septohippocampal co-cultures and hippocampal slices, 3–4 weeks of NGF stimulation is needed to observe changes in the neurotransmitter and in ChAT activity (52, 53). However, 4 weeks of *in vivo* treatment was insufficient to produce an increase in ACh. Thus, although we demonstrated increased levels of the metabolic enzymes by NGF, we did not find increased levels of ACh, suggesting another function of the cholinergic system not related to its action as a neurotransmitter and probably with trophic actions affecting follicular development.

### Unbalanced Intraovarian Cholinergic System and Over-Activated Sympathetic Pathway Due to Intraovarian NGF Excess Produced Altered Follicular Dynamics

Ovarian function was altered in rat ovaries exposed to NGF. The initial recruitment was altered because the number of secondary follicles was reduced, despite the fact that NGF promotes their growth and differentiation (54–56). Dissen et al. did not find changes in preantral follicles after grafting NGF-producing cells in rat ovaries. However, they were treated for 60 days with NGF, and compensatory mechanisms in initial recruitment could be responsible for their results (57). Regarding cycle recruitment, we found a decrease in the number of healthy antral follicles and an increase in atretic antral follicles. Since the GCs of healthy antral follicles express ChAT (21, 22), the reduction in their number could explain the reduction in acetylcholine levels after NGF treatment. The increase in atretic antral follicles could be an NGF-dependent atretic process mediated by its low-affinity NGFR (also termed as p75NTR) (58). However, NGFR is expressed at very low levels or is undetectable in the GCs of rat ovaries (59); therefore, the increment in atretic follicles may be promoted by other causes, such as necroptosis. Recently, Du et al. reported that the use of necrostatin, an inhibitor of a kinase of the necroptosis pathway, promotes the increase in the size of oocytes and follicles cultured *in vitro* (60). In this sense, the ARP fragment of AChE-R induces cell death activating kinases of the necroptosis pathway, and, as described above, induced by AChE-R, which thought that is the isoform incremented by our *in vivo* protocol. ARP induces necroptosis in human GCs (32). Hence, there is a relationship between the promotion of atresia by NGF and the increment in AChE-R and hence a decrease in ACh. Further research is needed to determine the mechanism involved in this process.

Cruz (5) suggested that ACh regulates ovulation through muscarinic receptors. We reported a decrease in the number of corpora lutea, specifically of newly formed (large) corpora lutea, and accumulation of small corpora lutea (data not shown). The increase in small corpora lutea is associated with a decrease in the number of ovulations. Moreover, NGF stimulates the formation of cystic structures. It is well known that cysts produce impairments in ovarian function, such as hyperandrogenism, anovulation, and infertility (12, 17, 51, 61). Overall, all these changes show that chronic NGF treatment produced a decrease in ovarian function, but not by a selective increase in noradrenergic tone but rather in both neurotransmitters, being a noradrenergic predominance because of the increase in the AChE degrading enzyme.

However, the present findings are not explained only by the hyperactivation of the sympathetic pathway in the ovary due to the increase in noradrenaline levels. Indeed, 4 weeks of cold stress treatment (chronic noradrenergic activation) increases ovarian noradrenaline and aberrant follicular development, but only it induces a decrease in number of secondary follicles, corpora lutea and appearance of precystic structures (6, 12, 62). Recently, it was described that CUMS chronic stress decreases NGF in the ovary (63). Probably is related to a non-specific increase in corticoids as it was demonstrated to occur in the ovary after a restraint/cold stress (61). The most important observation was that *in vitro* incubation with NGF reverse the changes induced by CUMS suggesting changes in the balance of the induced neurotransmitters (probably ACh) or in the inhibition of corticoids synthesis in the rats. Imbalance in the intraovarian cholinergic system may be the factor affecting normal follicular development, increasing ACh levels by inhibiting AChE-enhanced follicular development and reducing the number of cystic structures (4). Herein, the imbalance in ACh production/degradation favored its degradation. In addition, the increase in the AChE 55 kDa-isoform (probably AChE-R), which could produce inflammation directly or indirectly through ARP (which itself promotes necroptosis), could explain the more profound effects of NGF on aberrant follicular development (including the appearance of pre-cyst and cyst structures). These abnormalities with the over-activated sympathetic pathway may favor the promotion of the atretic pathway and cystic structures over healthy follicles.

In fact, if a decreased ovarian ACh concentration is the cause of the predominance of the noradrenergic tone responsible for the PCO phenotype, we obtained strong evidence of a regulating effect of ACh in the ovarian follicular development by the use of carbachol, a non-specific muscarinic agonist, which is not degraded by AChE. This drug produced clear changes in ovarian morphology, showing the results of a pure cholinergic effect; thus, the cholinergic stimulation led to an increase in the number of corpora lutea (i.e., ovulation). A decrease in cystic follicles indicates that cholinergic activation is in balance with sympathetic activation. These results are also supported by the experiments of Urra et al (4). in which they demonstrated that the increase in ACh in a normal ovary induced an increase in ovulation and fertility. In addition this is a characteristic of ACh because it is able to reverse the PCO phenotype induced by cold stress in rats (13).

Our results indicate that ACh is a key factor in follicular development, promoting the healthy pathway over the atretic or cystic pathway. The most important finding of this work is that NGF stimulates ovarian ChAT and VAChT mRNA and, probably, ACh production. *In vivo*, we found that NGF mainly induced the activation of the sympathetic pathway and increase in AChE 55 kDa-isoform, resulting in an imbalance in the ovarian cholinergic system and aberrant follicular development. Overall, it seems that NGF is a key factor in maintaining homeostasis in the dual autonomic control system, balancing the output of the sympathetic and cholinergic systems to regulate ovarian function. These results open the possibility to pharmacologically control the sympathetic and cholinergic activity by direct delivery of drug affecting the ovarian follicular development in the rat. These results needs to be validate in human ovary as it has accumulated for the noradrenergic control (7).

## Data Availability Statement

The raw data supporting the conclusions of this article will be made available by the authors, without undue reservation.

## Ethics Statement

The animal study was reviewed and approved by the Bioethics Committee of the Faculty of Chemistry and Pharmaceutical Sciences at the University of Chile (Protocol number: CBE2017-14 to AB and CBE2017-05 to HL). Sergio Livingstone 1007.

## Author Contributions

AB performed most of the experimental work with rats, biochemical analysis, data collection, and manuscript preparation. MdC performed estradiol studies. RR and CA performed the morphometric analysis in NGF and carbachol studies, respectively. HL conceived the idea, participated in the study design, and directed the work and manuscript preparation. All authors contributed to the article and approved the submitted version.

## Funding

This study was supported by grants from the Fondo Nacional de Ciencias Fondecyt 1170291 (to HL). AB was also supported by a scholarship for Doctoral thesis support Conicyt N° 21161218.

## Conflict of Interest

The authors declare that the research was conducted in the absence of any commercial or financial relationships that could be construed as a potential conflict of interest.
